# Editorial: Molecular mechanisms in diet-mediated inflammatory diseases

**DOI:** 10.3389/fnut.2023.1270271

**Published:** 2023-08-30

**Authors:** Beatrice Dufrusine, Michele Sallese, Enrico Dainese

**Affiliations:** ^1^Department of Bioscience and Technology for Food Agriculture and Environment, University of Teramo, Teramo, Italy; ^2^Department of Innovative Technologies in Medicine and Dentistry, University “G. d'Annunzio” of Chieti-Pescara, Chieti, Italy; ^3^Center for Advanced Studies and Technology, University “G. d'Annunzio” of Chieti-Pescara, Chieti, Italy

**Keywords:** nutrition, Westernized diet, Dietary inflammatory index (DII), immune system, inflammatory response

A balanced and varied diet is necessary for maintaining a healthy and functional state in all organisms. The dietary changes occurring in Western countries have determined an increase in the consumption of energy-dense, ultra-processed foods and a reduction of dietary fibers (1). These nutritional factors negatively affect human health and correspond to an increase in several inflammation-related pathological conditions, such as obesity (2), allergies (3), asthma (4), and cancer (5). Furthermore, dietary metabolites can modulate immune cells acting to reduce or delay the onset of immune-mediated diseases (6, 7). Although studies in animal and in cellular models have highlighted the close link between diet and immune homeostasis, molecular mechanisms by which dietary and primary metabolites interact with the immune system have been partially identified and require further studies.

Consequently, the title of this Research Topic “*Molecular mechanisms in diet-mediated inflammatory diseases*” was aimed to better clarify the link between the between dietary metabolites and immune cells and associated inflammatory diseases. Thus, we collected one Brief Research Report, one Clinical Trial, one review, and five original research articles by well-recognized authors on the complex relation between eating habits, nutrients and associated inflammatory diseases.

Dai et al. characterized the nutritional profile and the phytochemical compositions of *Adonis coerulea* and reported an anti-inflammatory/antioxidant activity at cellular level, thus underlining the important role of plants as a source of nutritional ingredients and natural agents to regulate immunity.

Wen et al. conducted a cross-sectional study composed of 28,605 participants to investigate the association of the Dietary Approaches to Stop Hypertension (DASH) diet with the risk of chronic obstructive pulmonary disease (COPD) in American adults, thus supporting the importance of diet in the pathogenesis of COPD.

Hu et al. investigated the impact of dietary pattern with high dietary inflammatory potential in cardiometabolic disease (CMD) in a cohort of 16,681 patients thus associating the Dietary inflammatory index (DII) with faster progression of the disease.

Yang et al. analyzed the genetic causal relationship between Ankylosing spondylitis (AS) and iron homeostasis-related biomarkers (ferritin, serum iron, total iron binding capacity TIBC, transferrin saturation TSAT) using Mendelian randomization and did not revealed a direct genetic causal relationship between AS and iron homeostasis.

Maestri et al. conducted a systematic review with the aim to highlighting a relationship between gut microbiota, non-alcoholic fatty liver disease, and possible therapeutic implications. They better clarified the conflicting scientific data regarding the gut microbiota due to the high complexity of the microbiota-host-external factors interaction.

Alhaj et al. proposed use of the Dietary inflammatory index (DII) as a predictor of Multiple sclerosis (MS) odds. They reported the association between diet rich in pro-inflammatory foods and the inflammation state and illness duration for 541 MS patients.

Li et al. conducted a large, prospective observational study composed of 127 participants with inflammatory bowel disease (IBD), aged 14–75 years. They examined the plasma lipidomic profile and reported a link between the abnormal lipid profile and leukopenia in patients with.

Dufrusine et al. evaluated the inflammatory effect of two commonly used dietary emulsifiers (EMs) on colon carcinoma cells and macrophages. They reported that EMs improved the inflammatory state by directly influencing the release of cytokines (IL-6 and CCL2) that in turn modulate the inflammatory state of macrophages.

In conclusion, these studies provide a number of interesting results and add new knowledge on the relationship between nutrition and several inflammatory diseases. We are grateful to “Frontiers in Nutrition” for giving us the possibility to propose this Research Topic and had contact with leading Authors in the field. We would like to thank the Researchers for adhering with their research to our Research Topic and the Reviewers for their time and suggestions, which improved the quality of the presented researches.

## Author contributions

BD: Writing—original draft. MS: Writing—review and editing. ED: Funding acquisition, Resources, Writing—original draft, Writing—review and editing.
